# Pten and EphB4 regulate the establishment of perisomatic inhibition in mouse visual cortex

**DOI:** 10.1038/ncomms12829

**Published:** 2016-09-09

**Authors:** Amy Baohan, Taruna Ikrar, Elaine Tring, Xiangmin Xu, Joshua T. Trachtenberg

**Affiliations:** 1 Department of Neurobiology, David Geffen School of Medicine at UCLA, Los Angeles, California 90095, USA; 2 Department of Anatomy & Neurobiology, UCI, Irvine, California 92697, USA

## Abstract

Perisomatic inhibition of pyramidal neurons is established by fast-spiking, parvalbumin-expressing interneurons (PV cells). Failure to assemble adequate perisomatic inhibition is thought to underlie the aetiology of neurological dysfunction in seizures, autism spectrum disorders and schizophrenia. Here we show that in mouse visual cortex, strong perisomatic inhibition does not develop if PV cells lack a single copy of *Pten*. PTEN signalling appears to drive the assembly of perisomatic inhibition in an experience-dependent manner by suppressing the expression of EphB4; PV cells hemizygous for *Pten* show an ∼2-fold increase in expression of EphB4, and over-expression of EphB4 in adult PV cells causes a dismantling of perisomatic inhibition. These findings implicate a molecular disinhibitory mechanism driving the establishment of perisomatic inhibition whereby visual experience enhances Pten signalling, resulting in the suppression of EphB4 expression; this relieves a native synaptic repulsion between PV cells and pyramidal neurons, thereby promoting the assembly of perisomatic inhibition.

## Abstract (web summary)

Fast spiking interneurons provide perisomatic inhibition in the cortex. Here, Baohan *et al*. show the failure to develop perisomatic inhibition in heterozygous *PTEN* null mutant mouse and the underlying mechanism to EphB4 reduction in an experience-dependent manner.

## Graphical abstract

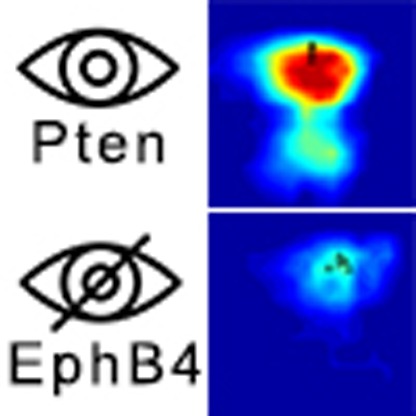

Perisomatic inhibition in cortex is established by fast-spiking interneurons called basket cells that express the calcium binding protein parvalbumin (PV). The establishment of this inhibition during postnatal development is centrally important to the opening of critical periods and the establishment of neural gain control, and its failure is linked to a number of disorders of cortical development, including autism and schizophrenia^1^,^2^,^3^,^4^. Remarkably little is known about how PV neurons establish strong synaptic input to local pyramidal neurons during development.

By comparison, a great deal more is known about the postnatal growth of pyramidal neurons. In mice, the growth of axons and dendrites is most pronounced during the first few weeks of postnatal life. Thereafter, growth is slowed as neurons begin to express PTEN^5^. Deleting *Pten* from adult pyramidal neurons is sufficient to re-start dendritic and axonal growth^5^,^6^,^7^.

Embryonic PTEN deletion from inhibitory neurons impacts the establishment of appropriate numbers of parvalbumin and somatostatin-expressing interneurons, demonstrating a central role for Pten in inhibitory cell development^8^. To examine how Pten signalling impacts PV cells specifically, we use loxP-mediated recombination to generate mice in which PV cells lacked one or both copies of *Pten*. Heterozygous animals (PV-*Pten*^+/−^) were studied exclusively, as complete knockouts died prematurely from seizures. Our studies focus on the primary visual cortex, where the development of perisomatic inhibition has been well characterized and is known to be strongly regulated by visual experience^9^,^10^,^11^. We find that single copy *Pten* deletion from PV cells impairs the formation of perisomatic inhibition. Pten expression in PV cells increases with visual experience, and Pten signalling appears to suppress the expression of the synaptic repulsive factor, EphB4, which is initially high in PV cells. Diminishing Pten expression either by dark rearing or by single copy gene deletion maintains elevated EphB4 expression, thereby repelling the establishment of PV cell synapses onto pyramidal neurons.

## Results

### *Pten* in PV cells regulates perisomatic inhibition

**Figure 1:**
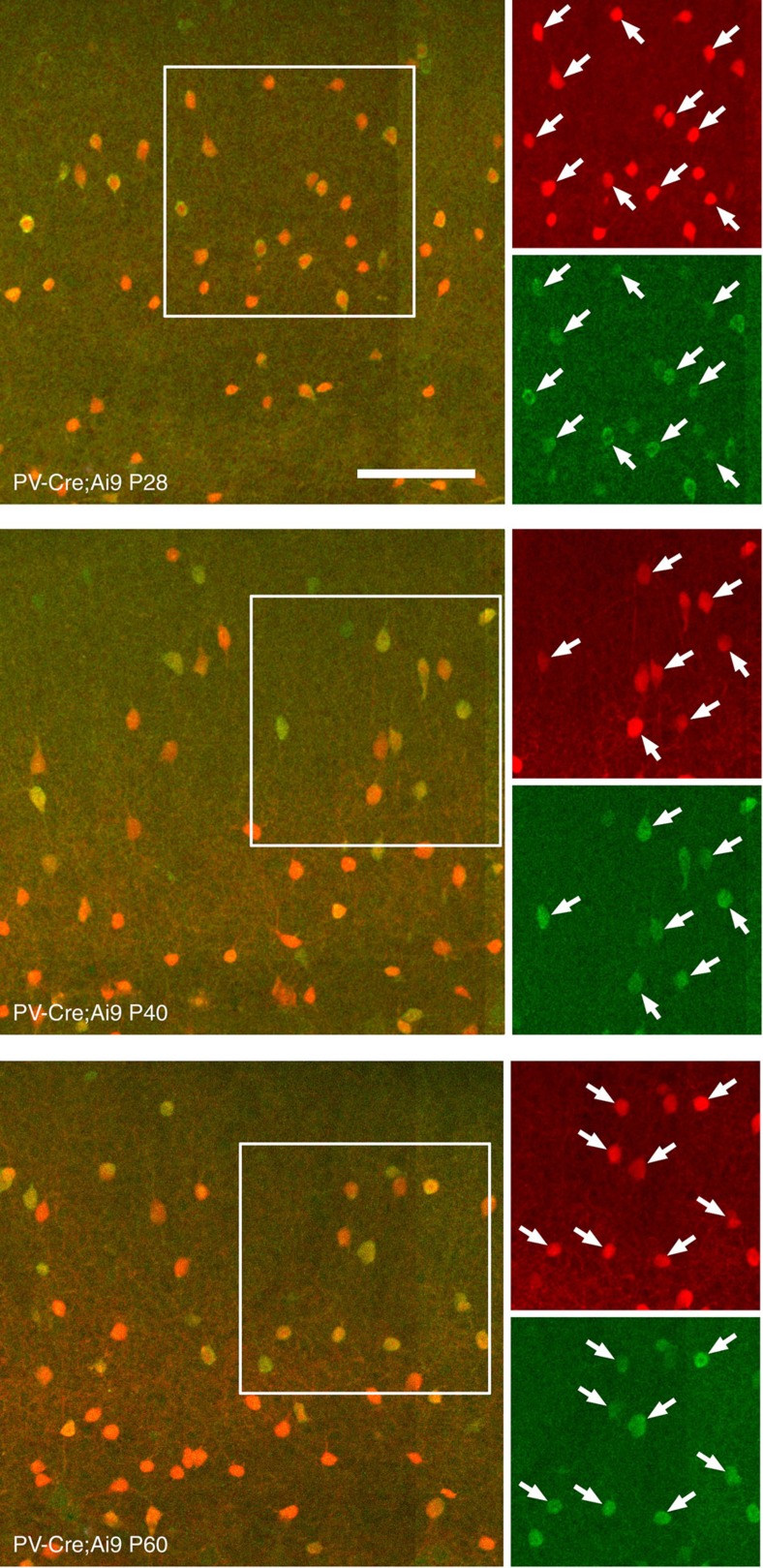
Expession of Cre recombinase in PV-Cre mice. Examples of expression of tdTomato (red) and parvalbumin (green) in primary visual cortex at three separate ages are shown overlaid on the large, left panel of each grouping. Higher magnification views of the region delineated by the white square are shown to the right. Arrows indicate cells expressing both tdTomato and parvalbumin. Ages are postnatal day 28 (top), P40 (middle) and P60 (bottom). Scale bar is 100 μm.

All studies were carried out using PV-Cre knockin mice obtained from The Jackson Laboratory (B6;129P2-*Pvalb*^*tm1(cre)Arbr*^/J). To validate the expression of Cre recombinase in these mice, we crossed them with the Ai9 line of reporter mice expressing a flexed tdTomato knocked in to the Rosa26 locus to generate mice expressing tdTomato in cells expressing Cre; the fraction of PV cells that also expressed tdTomato, and thus Cre recombinase in visual cortex, was then measured at three ages (postnatal days 28, 40 and 60) by immuno-staining against PV in fixed, frozen coronal sections. At P28, 92 of 93 PV cells counted in three sections (99%) were co-labelled; at P40 this ratio was 166/168 (99%), and at P60 it was 166/184 (90%), indicating that the PV-Cre line of mice reliably express Cre recombinase and tdTomato in virtually all PV cells (Fig. 1). These values are similar to those reported in ref. 12.

**Figure 2:**
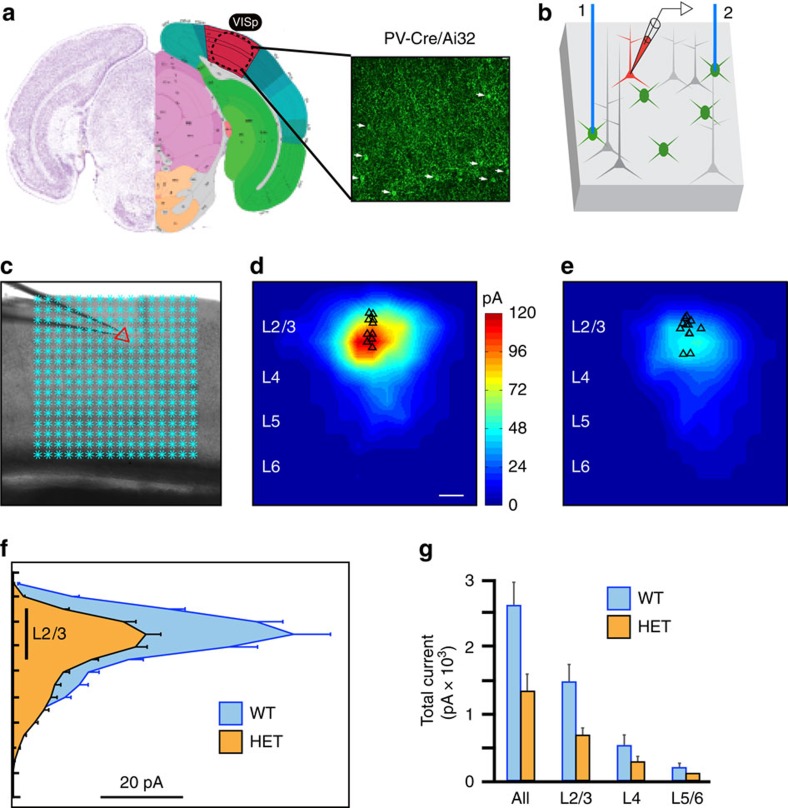
Reduced perisomatic inhibition following single copy *Pten* deletion from PV cells. (**a**) Brain section through visual cortex (delineated in red). The image to the right shows the pattern of YFP-tagged ChR2 expression in PV cells, achieved by crossing PV-Cre and Ai32 mouse lines. Image is not to scale; for illustrative purposes only. (**b**) Schematic of the ChR2-assisted circuit mapping approach. A pyramidal neuron (red) is recorded in voltage clamp as a blue laser tessellates the cortical slice in a pseudo-random 16 × 16 array, driving somatic spiking activity in ChR2-expressing PV cells. (**c**) Image of a cortical slice. The silhouette of a patch pipette is visible coming in from the left. The location of the patched pyramidal neuron is marked by the red triangle. Each blue asterisk identifies a unique spot of blue laser illumination. (**d**,**e**) Heat maps of inhibitory postsynaptic current strength and position averaged across 10 pyramidal neurons in wild-type mice (**d**, *n*=10 cells, 3 mice) and mice hemizygous for *Pten* in PV cells (**e**, *n*=12 cells, 3 mice). Scale bar in (**d**) is 100 μm. (**f**) Plot of laminar position (*y* axis) and summed postsynaptic inhibitory current (*x* axis), derived from the maps in (**d**,**e**). Note the significant loss of PV cell-derived inhibitory input to pyramidal neurons in PV-*Pten* heterozygous mice. (**g**) Total PV cell-mediated inhibitory currents from each layer to pyramidal neurons in cortical layer 2/3 in wild-type and PV-Pten heterozygous mice. Plots are mean +/− s.e.m. ** All *P*=0.01, L2/3 *P*=0.008; Kruskal-Wallis one-way ANOVA followed by Mann-Whitney *U*-test.

To examine the impact of *Pten* haploinsufficiency on PV cell circuitry, we expressed the light-activated cationic channel, channelrhodopsin-2 (ChR2), in PV cells of PV-*Pten*^+/−^ and littermate control mice; subsequently laser scanning photo-stimulation of ChR2-expressing PV cells with concurrent whole-cell recordings of layer 2/3 pyramidal neurons was used to measure the strength of PV cell input to pyramidal neurons in primary visual cortex in acute brain slices (P35-P56). We note that mapping studies based on ChR2 expression may be confounded by differences in the optical recruitment of PV cells. With this approach, we found that PV cells hemizygous for *Pten* produced only half of the total inhibitory drive to pyramidal cells of wild-type PV cells (Fig. 2; Supplementary Figs 1 and 2). This reduced inhibitory current could not be attributed to a reduced expression of ChR2 or responsiveness to blue light stimulation (Supplementary Fig. 3), or to changes in the intrinsic properties of PV cells in the experimental group (Supplementary Fig. 4), as these measures were comparable between PV cells hemizygous for Pten and controls. Nor was it due to a reduction in the number of PV cells in mutants (Cells per field of view visualized using a × 4 objective lens with a field number of 26.5; mean and standard deviation: WT=354 +/− 40; Pten=367 +/− 60; 10 fields of view per group; *P*=0.73, Wilcoxon rank sum).

**Figure 3:**
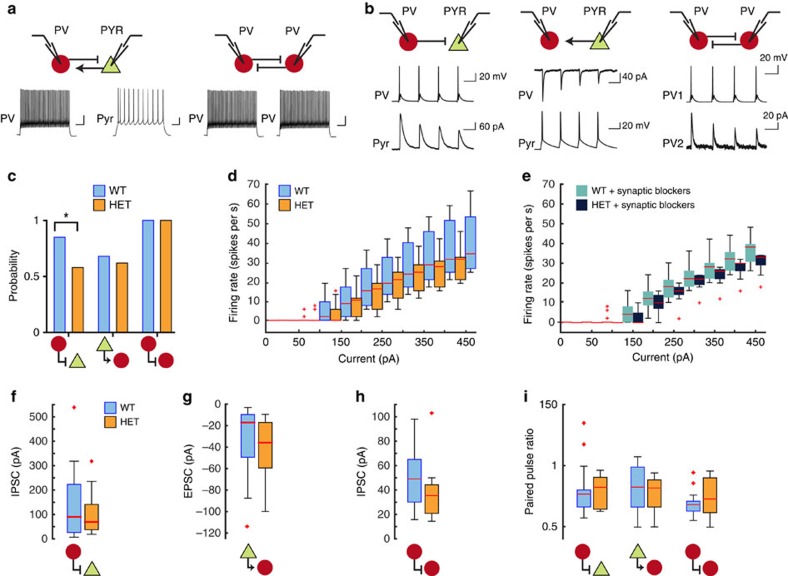
PV cells hemizygous for *Pten* establish fewer connections to local pyramidal neurons. (**a**) Examples of action potentials elicited in PV cells and pyramidal neurons during paired whole-cell recording experiments. Scale bars are 80 ms and 20 mV. (**b**) Examples of presynaptic action potentials and postsynaptic responses in paired recordings. All horizontal scale bars are 20 msec. (**c**) Probability of connection (ordinate) from PV cells to pyramidal cells in WT and PV-*Pten* heterozygous mice (*P*=0.025, Fisher's exact test; *n*=31 pairs in each genotype), pyramidal cells to PV cells (*n*=31 pairs from 10 mice in each genotype) and PV cells to neighbouring PV cells (*n*=33 pairs from 8 mice in each genotype). (**d**) Plot of evoked firing rates of pyramidal neurons to varying amounts of current injection in control, PV-*Pten* heterozygous mice (WT=10 cells, 3 mice; HET=10 cells, 3 mice; *P*=0.003, Kruskal-Wallis one-way ANOVA, followed by individual rank sum tests. *P*=0.02 for comparisons at 150 pA. For all other comparisons, *P*>0.05). (**e**) Same plot as in **d**, but following bath application of APV, picrotoxin and CNQX (WT=15 cells, 3 mice; HET=12 cells, 3 mice; *P*=0.92, Kruskal-Wallis one-way ANOVA). (**f**) Plot of amplitude of inhibitory postsynaptic currents recorded in pyramidal cells upon stimulation of presynaptic PV cells (WT=24 pairs, 11 mice; HET=13 pairs, 5 mice; *P*=0.69, Wilcoxon rank-sum test). (**g**) Amplitudes of excitatory postsynaptic currents recorded in PV cells upon stimulation of presynaptic pyramidal neurons (WT=20 pairs, 10 mice; HET=19 pairs, 6 mice; *P*=0.07, Wilcoxon rank-sum test). (**h**) Amplitudes of inhibitory postsynaptic currents recorded in PV cells upon stimulation of presynaptic PV cells (WT=20 pairs, 6 mice; HET=9 pairs, 3 mice; *P*=0.19, Wilcoxon rank-sum test). (**i**) Paired pulse ratios recorded in each of the three types of connections. All *P*-values >0.05, Wilcoxon rank-sum test. Plots in **d**–**i** are box plots. Red crosses identify major outliers.

This reduction in PV cell-derived inhibitory current could result from pyramidal neurons receiving fewer PV cell-derived inhibitory synapses; these synapses could be present in normal numbers, but weaker synapses; or it could be some combination of these two. If there are fewer synapses, this would be reflected in a decrease in connectivity. If the synapses are weaker, this would be reflected in a decrease in paired-pulse ratio. To directly measure these, we conducted paired whole-cell recordings from adjacent PV cells and pyramidal neurons and, separately, from adjacent pairs of PV cells (Fig. 3). Recordings were done in L2/3 of the primary visual cortex of young adult mice (P35–56). PV cells were identified by their expression of td-Tomato, high frequency firing pattern, narrow action potentials and sharp after-hyperpolarizations. Pyramidal neurons were identified by their lack of td-Tomato expression and the presence of an accommodating firing pattern (cf. Fig. 3a). Connectivity between the two cells was assessed by stimulating the presynaptic neuron in current clamp and recording from the putative postsynaptic neuron in voltage clamp (cf. Fig. 3b). PV cells hemizygous for *Pten* exhibited a 32% decrease in probability of connecting to adjacent pyramidal neurons relative to controls (Fig. 3c). This 32% reduction is a measure of connectivity and is distinct from the 50% reduction in total PV cell-derived inhibitory current in pyramidal neurons reported in Fig. 1, which is a measure of total input strength. Notably, the probability of finding the reverse connection, from pyramidal cell to PV cell hemizygous for Pten was no different from control; nor was there any change in the probability of connection between mutant PV cells and between normal PV cells (Fig. 3c).

When we examined the intrinsic excitability of pyramidal neurons in these slices, we found a trend towards increased excitability in PV-*Pten*^+/−^ mice (Fig. 3d; only comparisons at 150 pA had a *P* value <0.05, no other comparisons revealed significant differences). This data trend could be due to a true change in the intrinsic properties of these excitatory neurons, despite having normal *Pten* copy number, or it could simply be an epiphenomenon of the reduction in inhibition in these slices, one result of which would be an increase in recurrent excitatory response following the firing of the recorded excitatory neuron. Supporting this latter view, the data trend towards increased excitability was erased when all synaptic transmission was blocked by application of AMPAR, NMDAR and GABA_a_R antagonists (CNQX, APV, picrotoxin; Fig. 3e; *P*=0.92, Kruskal-Wallis one-way analysis of variance (ANOVA)). These paired recordings show that PV cells hemizygous for *Pten* have normal intrinsic membrane properties, but establish fewer synapses onto local pyramidal neurons.

To determine whether the synapse that are established by the mutant PV cells are of normal strength, we measured response amplitudes and paired pulse ratio of extant connections. We found no significant differences in response amplitudes in any of the types of synapses examined; PV to pyramidal, pyramidal to PV, and PV to PV were all comparable between PV-Pten^+/−^ mice and controls (Fig. 3f-h). Measures of paired pulse ratio were also equivalent (Fig. 3i). These measures indicate that the synapses established by mutant PV cells onto local pyramidal neurons are as strong as synapses established by normal PV cells.

Taken together, these findings show that single copy *Pten* loss in PV cells reduces the probability that any given PV cell will establish a connection with a local pyramidal neuron; this, in turn, causes a significant reduction in the strength of perisomatic inhibition. This effect is specific to the connection between PV cells and pyramidal cells.

### Mutant PV cells express high levels of EphB4

**Figure 4:**
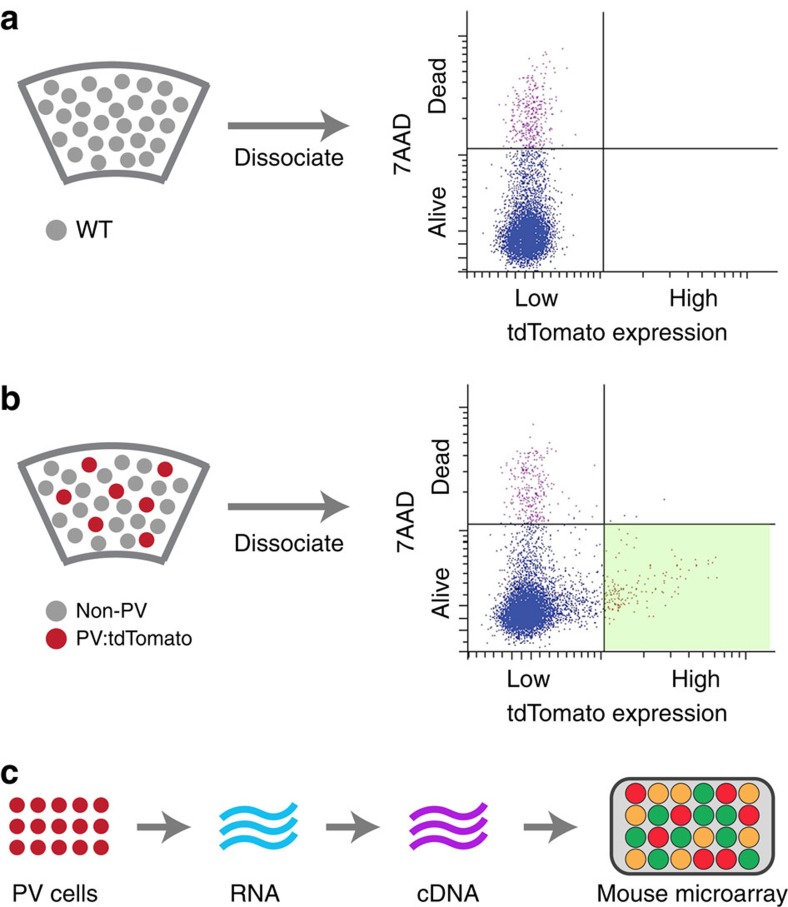
PV cells hemizygous for Pten have increased EphB4 expression. (**a**) Left: schematic of a slice through visual cortex. Right: FACS signals for cells dissociated from cortex in a WT mouse with no tdTomato expression in any cortical neurons. Ordinate: log scale of brightness of 7AAD fluorescence staining; Horizontal line defines boundary used to separate live from dead cells. Abscissa: log scale of tdTomato fluorescence; Vertical line defines the boundary, later used in panel (**b**) to distinguish tdTomato-expressing cells. (**b**) Example of FACS signals for cells dissociated from cortex in a PV-Cre/Ai9 mouse expressing tdTomato in PV cells. The lower right quadrant identifies the levels of 7AAD staining and tdTomato expression used to separate living, tdTomato-expressing PV cells. (**c**) Schematic of the process used to create and quantify cDNA libraries from PV cells in normal mice and mice hemizygous for *Pten* in PV cells. FACS, fluorescent-activated cell sorting.

We next examined how a reduction in Pten expression could result in decreased, synapse-specific connectivity between PV cells and pyramidal neurons. Since PTEN is a phosphatase that negatively regulates the AKT signalling pathway, which in turn regulates gene transcription and protein translation^13^, we screened for possible differences in gene expression in *Pten*^+/−^ and wild-type PV cells. To do so, live PV cells were harvested from the primary visual cortex of intact adult brains via fluorescence-assisted laser cell sorting (Fig. 4a,b). RNA from live PV cells was isolated and amplified, and complementary DNA libraries synthesized. Transcript copy number was quantified using Illumina mouse gene chip arrays, with a total of 26,500 genes screened (Fig. 4c). Seventeen transcripts met established criteria for further examination, as described in ref. 14, namely a >1.4-fold change in expression (logFC>0.5) and a *P*-value <0.001 (Table 1). Of these, the majority encoded intracellular glycosylation proteins. One standout transcript was *EphB4* (logFC=0.56, *P*=0.00039), which encodes the presynaptic partner of ephrinB2^15^. Quantitative real-time PCR (ddCt=1.84) confirmed that *Ephb4* RNA was in fact increased in PV cells hemizygous for *Pten*.

These data suggest a model in which Pten expression in PV cells suppresses expression of EphB4 in these same cells. In the central nervous system, ephrinB/EphB signalling mediates cell-to-cell repulsion during development^16^,^17^,^18^. Supporting this view, RNA sequencing data of adult mouse primary visual cortical neurons, published from the Allen Brain Institute, shows high expression of EphrinB2 in pyramidal neurons; Pten, but not EphB4 is expressed in adult PV cells (see http://casestudies.brain-map.org/celltax). Increased expression of EphB4 in PV cells could account for the reduction in PV to pyramidal inhibitory synapses via increased repulsion between the presynaptic terminals of PV cells and cell bodies of pyramidal neurons.

### EphB4 overexpression dismantles perisomatic inhibition

**Figure 5:**
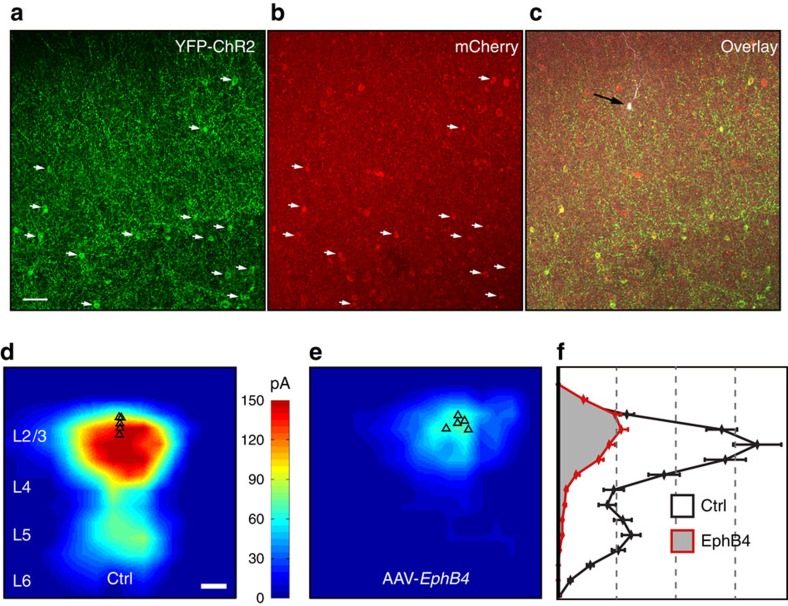
EPHB4 over-expression in PV cells reduces perisomatic inhibition of pyramidal neurons. (**a**) Representative image of a cortical slice from a PV-Cre/Ai32 mouse showing YFP-tagged ChR2 expression in PV cells. White arrows in this panel and the next show the positions of PV cell somas. Scale bar for panels **a**–**c** is 50 μm. (**b**) mCherry expression in the same field of view as in **a**. Here, mCherry is expressed in cells that also express *Ephb4*, as the same viral vector expressed both genes: AAV2/1-Syn.flex.mCherry.D2A.mEphB4. Note that the white arrows in **a**,**b** show that YFP and mCherry, and by extension ChR2 and EPHB4, are co-expressed in PV cells. (**c**) The black arrow and white cell body identify the position of a L2/3 pyramidal neuron patched in this slice and recorded during ChR2-assisted circuit mapping of PV cell inputs. (**d**,**e**) Aggregate heat map of the spatial spread and strength of PV cell input to L2/3 pyramidal neurons in wild-type mice (**d**, *n*=5 cells, 2 mice) and mice over-expressing *Ephb4* in PV cells (**e**, *n*=5 cells, 2 mice). Scale bar is 200 μm. (**f**) Laminar distribution of average PV cell input derived from the heat maps in **d**,**e**. Separation between dashed lines is 20 pA. Plots are mean +/− s.e.m. Kruskal-Wallis one-way ANOVA.

To directly test whether increased EphB4 signalling in PV cells is sufficient to prevent the normal formation of perisomatic inhibition of pyramidal neurons, we selectively over-expressed *Ephb4* in mature, genotypically normal PV cells in the primary visual cortex of mice expressing ChR2 under the PV promoter (PV-Cre/Ai32) via viral transduction of *Ephb4* (AAV2/1-Syn.flex.mCherry.D2A.mEphB4; Fig. 5a-c) and then mapped net PV cell-mediated inhibition across all cortical layers onto individual pyramidal neurons via laser scanning photo-stimulation of ChR2 expressing PV cells with concurrent whole-cell recordings of pyramidal neurons as in Fig. 1. Control recordings were obtained from PV-Cre/Ai32 mice that received the same viral injection but failed to express *Ephb4*. A 67% reduction in local PV mediated inhibition of pyramidal neurons was observed in mice overexpressing *Ephb4* (Fig. 5d-f), supporting the view that EphB4 signalling in PV cells impairs the formation of perisomatic inhibitory synapses.

### Early vision increases Pten and suppresses EphB4 in PV cells

**Figure 6:**
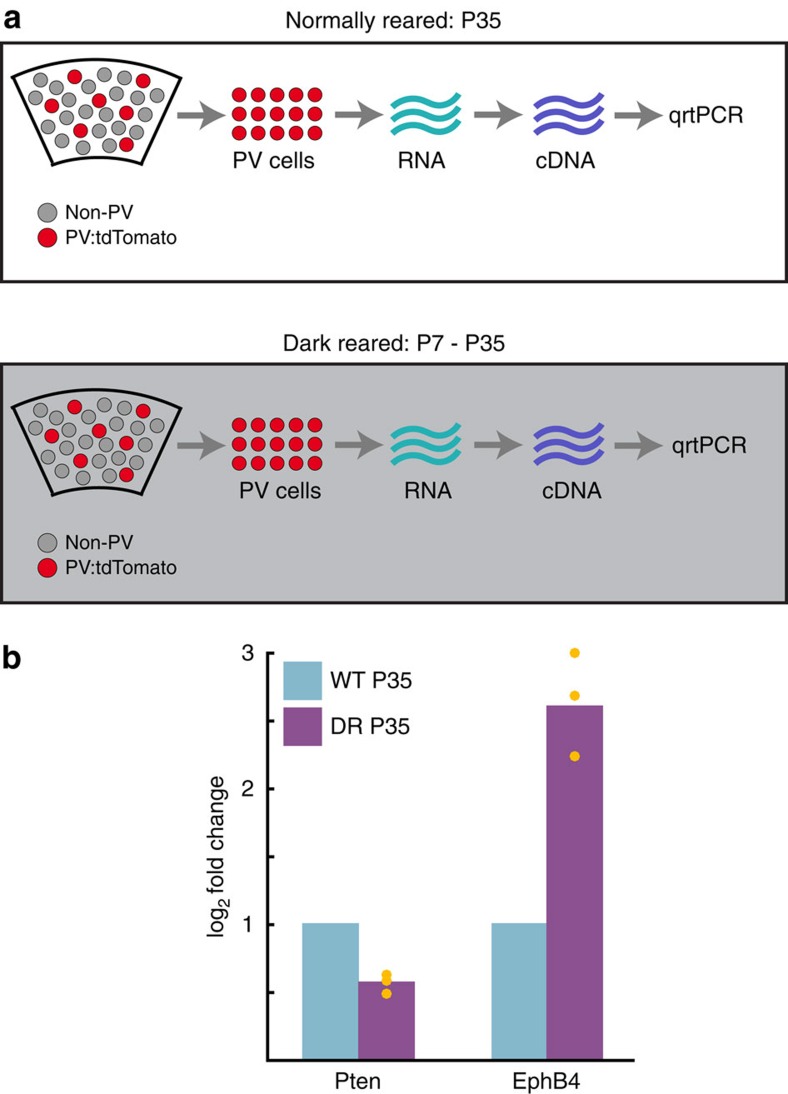
Visual experience regulates Pten and EphB4 mRNA levels in PV cells. (**a**) Schematic overview of the approach taken to isolate live PV cells from primary visual cortex in mice raised with normal vision and in mice raised in the dark from postnatal day 8 to postnatal day 35. PV cells expressing tdTomato were fluorescently sorted, live cells were selected, RNA libraries were constructed, cDNA libraries were made, and the expression Pten and EphB4 message was quantified using real-time PCR. (**b**) Plot of log2 fold change in expression of Pten and EphB4 in PV cells. All values were normalized to the wild-type values. Note the decrease in Pten and increase in EphB4 in the absence of vision. Mean values are Pten dark reared: 0.56, EphB4 dark reared: 2.65. Orange circles are values of individual mice (*n*=3 mice, >40,000 PV isolated from each mouse).

Since visual experience is required for the formation of perisomatic inhibition in visual cortex, we wondered whether vision also informs levels of Pten and EphB4 expression in PV cells. To do so, we reared PV-Pten^+/−^ mice and littermate controls in darkness from birth until postnatal day 35. PV cells in these mice also expressed the red fluorescent protein tdTomato. Using fluorescent-assisted laser cell sorting to extract live PV cells and subsequent qrtPCR normalized to GAPDH expression, we found a ∼3-fold increase in EphB4 expression in PV cells from dark reared mice relative to normally reared controls, and an ∼40% reduction in Pten expression (Fig. 6). These data support a model in which visual experience, at least in part, regulates the expression of EphB4 and Pten in primary visual cortex.

## Discussion

The establishment of perisomatic inhibition progresses through two stages. In the first stage, factors intrinsic to developing cortex guide PV axons to pyramidal cell somas and proximal dendrites^19^. In the second stage, increased neural activity incited by sensory experience drives the proliferation of perisomatic inhibition^20^. Our results suggest a model in which sensory experience suppresses expression of EphB4 in PV cells. This downregulation of EphB4 likely relieves a native synaptic repulsion between PV cells and pyramidal neurons, thereby promoting the assembly of PV-mediated perisomatic inhibition.

The link between increased Pten expression and a reduction in EphB4 levels has been shown in human tumour lines^21^, but has not been studied in the central nervous system. The signalling pathway linking Pten expression to a reduction in EphB4 expression is not revealed in this study, and the complexity of protein social networks, which are quite large, almost certainly guarantees that the connection will not be linear. However, Pten is known to suppress transcription factors. In mouse cortex, Pten expression increases approximately one week after birth and its expression increases through the third postnatal week^22^. In pyramidal neurons, Pten signalling slows the growth of axons and dendrites, and its deletion induces their growth^5^,^6^. Disruptions of the Pten signalling pathway in excitatory neurons are linked to autism spectrum disorders^23^, and, targeted, conditional deletion is being exploited as a strategy for promoting neural regeneration in the central nervous system^7^,^24^. Because of its link to autism and its potential therapeutic use in neural regeneration, PTEN signalling has been extensively studied in pyramidal neurons. However, PTEN's influence on the development of inhibitory neurons has only recently been examined^8^. This despite evidence that autism and schizophrenia may be deeply rooted in deficits in inhibition^4^,^25^,^26^. The data we present here show that in PV cells, *Pten* deletion promotes the disassembly of their synaptic input to local pyramidal neurons. This effect is specific to these synapses, as we found no evidence for changes in excitatory input to mutant PV cells or in inhibitory connections between mutant PV cells. These results, along with those of other investigators^8^, identify an essential link between PTEN signalling and the establishment of somatic inhibition.

## Methods

### Animal research

All experimental procedures were approved by the University of California Los Angeles Office for Protection of Research Subjects and the Chancellor's Animal Research Committee and by the Office of Research of the University of California, Irvine.

### PV-Cre^+/−^/Ai9^+/−^/*Pten* ^loxP+/−^ mice

To genetically label PV-positive neurons, PV-IRES-cre knock-in female mice (Jackson Laboratories, stock#008069, generated by S Arber, FMI^27^) were crossed with male TdTomato reporter knock-in mice (Jackson Laboratories, stock #007905, ‘Ai9', generated by H Zeng, Allen Brain Institute^28^). Offspring were hemizygous for both transgenes. These mice were then back-crossed with PV-Cre mice to generate offspring that were homozygous for PV-Cre and hemizygous for Ai9. Using separate breeders, we also generated mice that were hemizygous for both Ai9 and a floxed Pten^29^. PV-Cre^+/+^/Ai9^+/−^ mice were then crossed with Ai9^+/−^/Pten^loxP+/−^ mice to generate offspring hemizygous for Cre, Ai9 and Pten in PV cells. Littermate controls were hemizygous for Cre and Ai9 in PV cells, but expressed both copies of Pten.

### PV-Cre^+/−^/Ai32^+/−^/*Pten* ^loxP+/−^ mice

To generate mice in which a single copy of *Pten* was deleted from PV cells, we first crossed *Pten*^loxP+/−^ mice with mice homozygous for a conditional knock-in allele of ChR2-YFP (Jackson Laboratories, stock #012569, ‘Ai32', generated by H Zeng, Allen Brain Institute^30^). Resulting offspring were genotyped and mice hemizygous for ChR2-YFP and *Pten*^loxP+/−^ were crossed with PV-Cre^+/+^/Ai32^+/−^ mice to generate PV-Cre^+/−^/Ai32^+/−^/*Pten*^loxP+/−^ mice expressing a YFP-tagged ChR2 in PV cells that were either hemizygous for *Pten* (experimental) or expressed both copies of *Pten* (littermate controls).

### ChR2-assisted circuit mapping

Briefly, whole-cell recordings were obtained from pyramidal neurons in layer 2/3 of the primary visual cortex of PV-Cre^+/−^/Ai32^+/−^/*Pten*^loxP+/−^ mice and littermate controls. Spatial maps of PV connectivity strength to each patched pyramidal neuron were derived by systematically stimulating ChR2-expressing PV cells at 256 different sites arranged in a 16 × 16 matrix spanning all cortical layers^31^. Spatially restricted optogenetic activating of ChR2-expressing PV cells was accomplished using a 473 nm blue laser (30 mW, 0.25 ms). Voltage and current clamp controls were performed to normalize light stimulation protocols between slices and across animals. During optogenetic stimulation experiments, the ionotropic glutamate receptor antagonists (10 μM CNQX and 5 μM CCP) were added to the bath solution to block excitatory synaptic input (GABAergic transmission is unaffected) and avoid any potential dis-inhibition effects. Because interneurons can also be connected by electrical synapses, we blocked gap junctions using 100 μM carbenoxolone. Whole-cell voltage-clamp recordings were made from the recorded pyramidal neurons to measure photoactivation-evoked inhibitory postsynaptic currents at the empirically determined holding potential at +5 mV in voltage clamp mode with cesium-containing internal solution.

We validated the effectiveness of our approach as follows: following sufficiently high ChR2 expression in the cortex of the cross-bred pups (PV-Cre:Ai32), living brain slices were prepared and whole-cell recordings were obtained from ChR2/YFP-expressing PV cells that exhibited robust photoactivation-evoked spikes to repeated laser flashes (473 nm, 30 mW). To map their local functional outputs, whole-cell recordings from targeted excitatory and inhibitory neurons were combined with focal photoactivation (laser spot diameter, ∼50 μm) of ChR2+ inhibitory neurons to assess functional connectivity and synaptic properties. As ChR2-expressing PV cells only fire action potentials to photoactivation (0.25 ms, 30 mW) close to the cell body, we found that ChR2 photoactivation had sufficient spatial precision for laminar circuit mapping.

For ChR2-evoked PV+ synaptic input analysis, we used the 3 ‘hit' criterion, and only map locations with three detected inhibitory postsynaptic currents (IPSCs) were selected for further map construction (Supplementary Fig. 1). The IPSC peaks were detected within the window of between 3 and 50 ms to each laser flash, with a threshold determined by background spontaneous IPSCs. The average IPSC input amplitude/strength of each stimulation site was measured by normalizing the sum of individual IPSCs from each photostimulation site by the analysis durations; this average integrated value was expressed in pA. We quantified the total PV+ IPSC inputs (total current) for the recorded individual cells by summing the input values across all map locations. The individual maps from different neurons were aligned to generate group averaged maps, using the 4 × bright field images that were acquired during experiments to guide and register photostimulation sites. We used laminar cytoarchitectonic landmarks as well as the pial surface and white matter. These routine processes have been described in ref. 32.

### Paired patch recordings

Brains were dissected from adult (P35-56) male and female mice. Three hundred and fifty micrometre coronal cortical slices were cut containing the visual cortex. All recordings were done at 33–35 °C. Recordings were made in L2/3 at depths greater than 50 μm from the surface of each slice. PV cells were identified by their td-Tomato expression as well as their unique firing pattern, characterized by narrow action potentials, sharp after-hyperpolarization and fast firing rate. Pyramidal neurons were identified by their lack of td-Tomato expression, as well as their accommodating and slower firing pattern. Only cells with access resistance <25 MΩ were included in the subsequent analysis. Initial assessment of firing pattern was done in current clamp (IC) at resting membrane potential with current injections at 50 pA increments. Assessment of connectivity between pairs was done with the presynaptic cell in current clamp (VC) and the putative postsynaptic cell in VC.

### Fluorescent-activated cell sorting

Fluorescent-activated cell sorting was performed at the UCLA Flow Cytometry Core using a FacsARIA cell sorter. In brief, 500 μm thick coronal slices containing the visual cortex were cut in standard artificial cerebrospinal fluid (ACSF). A Worthington Biochemical Papain Dissociation kit was used to dissolve the extracellular matrix to obtain individual cells, which were then filtered through a 70 μm diameter mesh and labelled with the dead cell stain 7AAD. Cells were laser sorted for peridin-chlorophyll protein complex (PerCP) signals (7AAD) and phycoerythrin (PE) signals (td-Tomato). Cells from wild-type animals with no fluorescent proteins were used to calibrate PerCP and PE signals each time. Cells with high PE signals and low PerCP signals were collected. We looked to obtain greater than 60,000 live PV cells per sorting session.

### RNA amplification

RNA from collected PV cells was extracted with the Qiagen RNeasy Mini kit and then stored at −80 °C. RNA quality and quantity were assessed with the Bioanalyzer (Agilent). Only samples with RNA Integrity Number >8 were subsequently used. cRNA was then generated and amplified with the NuGEN Ultra Low Mass kit (Ovation).

### Microarray

Microarray experiments were performed at the UCLA Neuroscience Genomics Core. cRNA levels were measured using Illumina mouse arrays (MouseWG-6 v2.0 BeadChip). Identification of differential gene expression was accomplished by first normalizing the gene expression of each sample and then comparing the gene expression of *Pten*^+/−^ PV cells with that of wild-type (PV-Cre: Ai9) brains. Differential expression analysis was determined by calculating the log2 fold change (logFC), defined as the ratio of intensities between the two experimental conditions under comparison. For all comparisons, a gene was considered differentially expressed if it had a corrected *P*-value≤0.001, and a logFC⩾0.5 (⩾ 1.4-fold increased or decreased expression).

### qrt-PCR

Real-time quantitative PCR of the same cRNA libraries was used to validate hits (here, *Ephb4*). Approximately 300–500 ng of RNA from the amplified control and PTEN+/− samples was set aside prior to the microarray experiments. Thus, qrtPCR was performed on the same samples used in the microarray experiments. Total RNA from each sample was treated with DNAse 1 and converted into cDNA. Assays were performed in triplicate and analysed using an ABI 7700 instrument. The fold change was calculated using both standard curve analysis and the Pfaffl method.

### Immunostaining

P16-P42 mice were perfused with 1 × PBS followed by 4% PFA. Brains were removed and stored in 4% PFA overnight and transferred to a 30% sucrose solution with 0.05% NaN3 prior to cryosectioning. Brains were cryosectioned in the sagittal or coronal plane through primary visual cortex at 50 μm thickness per section. Free-floating sections were washed three times with 1 × PBS prior to being placed in a 2 N HCl solution for 17 min for antigen retrieval of EphB4. They were subsequently washed three times in 1 × PBS and blocked with 15% Normal Goat Serum in 0.5% Triton for 1.5 h at room temperature. The sections were then placed in a solution containing a monoclonal mouse anti-EphB4 primary antibody (1:50, Invitrogen) with 0.5% Triton overnight on a shaker in 4 °C. The following day, slices were washed and incubated with an Alexa488-conjugated goat anti-mouse secondary antibody (1:1000, Invitrogen) for 2.5 h at room temperature on a shaker.

### AAV-EphB4 virus injection

Through a 2.5 mm craniotomy centred on primary visual cortex, we made 3–4 injections of AAV2/1-e/Syn.FlexON.mCherry-T2AmEphB4 (1.2 × 10̂13 GC/ml). Injection sites were roughly 200 μm apart (forming a triangle with three injection sites or a square with four injection sites). Thin walled glass pipettes were pulled to have a long tapered tip with an opening that is <1 μm wide. The glass pipette was filled with ∼2 μl of virus and the tip of the pipette was gently broken using a Kimwipe to allow a small drop of virus to push through. The pipette was lowered to a depth of 300 μm below the surface of the cortex. Using a pressure of 15–20 psi, 1 msec puffs of virus were given 40–50 times with 2 s separating each puff. Then, the pipette was raised 50 μm towards the surface of the cortex and again 30–40 puffs were injected. This was repeated for every 50 μm up to 100 μm below the pial surface. Mice were euthanized 3 weeks post injection to allow sufficient time for EphB4 expression. Cortical slices prepared and ChR2-assisted circuit mapping was conducted as above.

### Statistical analyses and graphs

All data are reported as mean±standard error of the mean, except for data in Fig. 3c where the probability of connection is reported between patched pairs of cells. Significance in Fig. 3c was determined using Fisher's exact test. For the remaining data sets, when comparing two independent groups, a Wilcoxon rank-sum test was used. In the case more than two independent groups were compared a Kruskal-Wallis one-way ANOVA was used and followed by *post-hoc* comparisons when justified (alpha set to 0.05) using Wicoxon rank-sum tests. In the cases where more than two groups were compared and the groups were not independent, a repeated measures ANOVA (Friedman's test) was used. In all cases, sample size is defined as cell number.

### Data availability

All analyses were conducted in Matlab (Mathworks, Natick, MA). The code and data are available on request from the authors

## Additional information

**How to cite this article:** Baohan A. *et al*. Pten and EphB4 regulate the establishment of perisomatic inhibition in mouse visual cortex. *Nat. Commun.* 7:12829 doi: 10.1038/ncomms12829 (2016).

## Supplementary Material

Supplementary Information Supplementary Figures 1-4

## Figures and Tables

**Table 1:** List of non-metabolic genes differentially expressed in PV cells hemizygous for *Pten*.

**Gene**	***P*****-value**	**Fold change**	**Validation**
CD300A	5.71E-5	1.484	
E030006K04RIK	0.000182	1.641	
ANLN	0.000374	0.597	
**EPHB4**	**0.000392**	**1.475**	**q-RT PCR**
VLDLR	0.000577	0.730	
BIN1	0.000585	0.588	
GJA5	0.000621	1.306	
STMN4	0.000661	0.544	
EVI1	0.000676	1.591	
ADRA2C	0.000683	0.707	
9930117H01RIK	0.000703	1.575	
TGFBR1	0.000880	0.745	
CEACAM1	0.000885	1.351	
APOE	0.000908	1.301	
VLDLR	0.000921	0.683	
BCL2L2	0.000991	0.781	
OLFR6	0.000999	1.345	

Criteria for inclusion were a logFC>0.5 and *P*<0.001 (see ref. 28). Note the increased expression of *Ephb4*. Real-time quantitative PCR (ddCt-1.84) was used to verify this result.
