# Supplementary material for: Pten and EphB4 regulate the establishment of perisomatic inhibition in mouse visual cortex
Source: Nat Commun. 2016 Sep 9;7:12829. doi: 10.1038/ncomms12829 (PMC5023968; doi:10.1038/ncomms12829)
Supplement: Supplementary Information — Supplementary Figures 1-4 [file ncomms12829-s1.pdf]

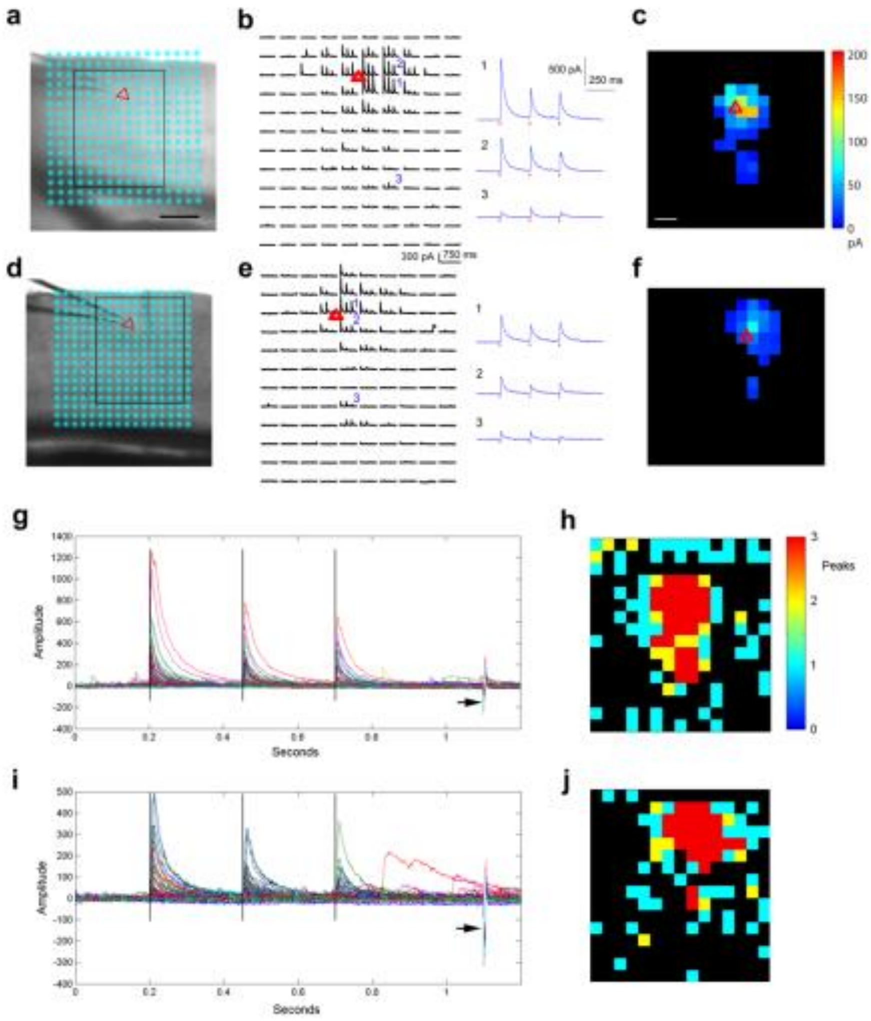

Supplementary Figure 1. Optogentic stimulation mapping of PV cell-derived inhibitory inputs to layer 2/3 pyramidal neurons.

a-c. PV cell inputs to a L2/3 excitatory cell of a WT slice. a shows a slice image with superimposed photoactivation sites (cyan *). Scale = 250 µm. The recorded excitatory cell location is shown in a red triangle.

b shows raw response traces of the recorded cell through focal photoactivation of ChR2-expressing PV+ inhibitory cells at different sites in the marked region in **a**. The responses are plotted for 750 ms beginning at the first of repeated laser flashes. The insert shows example inhibitory postsynaptic currents (IPSCs) detected in the recorded neuron to repeated laser flashes (0.25 ms. 30 mW, 250 ms interval; indicated by the short red ticks beneath the traces) at the specified sites. Only the sites with 3 stimulation-time locked, robust responses are included for further input analysis (see g-h below).

c shows the raw response maps as color-coded input maps.

d-f. PV+ inputs to a L2/3 excitatory cell of a PTEN slice, similarly formatted as in **a-c**. Note the clear reduction of PV+ IPSC inputs in the mutant slice.
g. The plot of all IPSC responses from the raw response shown in b with baseline subtraction. The three vertical black lines denote the laser stimulation onsets at 200 ms, 450 ms and 700 ms. The recoding access resistance was monitored by a current injection response (5 pA, 5 ms) shown by the arrow.
h. The map coding locations with different numbers of detected IPSCs in response to 3 repeated optogenetic stimulation per location. IPSC peaks are detected within the window of between 3 - 50 ms to each laser flash, with an empirically determined threshold of 20 pA. The threshold matches the average spontaneous IPSC amplitude shown in g. Only sites with 3 detected IPSCs are selected for further map construction.
i. The plot of all IPSC responses from the raw response shown in e with baseline subtraction. The recoding access resistance was monitored by a current injection response (5 pA, 5 ms) shown by the arrow.
j. The map coding locations with different numbers of detected IPSCs in response to 3 repeated optogenetic stimulation per location. IPSC peaks are detected within the window of between 3 - 50 ms to each laser flash, with an empirically determined threshold of 20 pA.
Scale bar in **c** also applies to panel **f** and is 120 µm. This scale indicates the averaged integrated IPSC input amplitude at each map site.

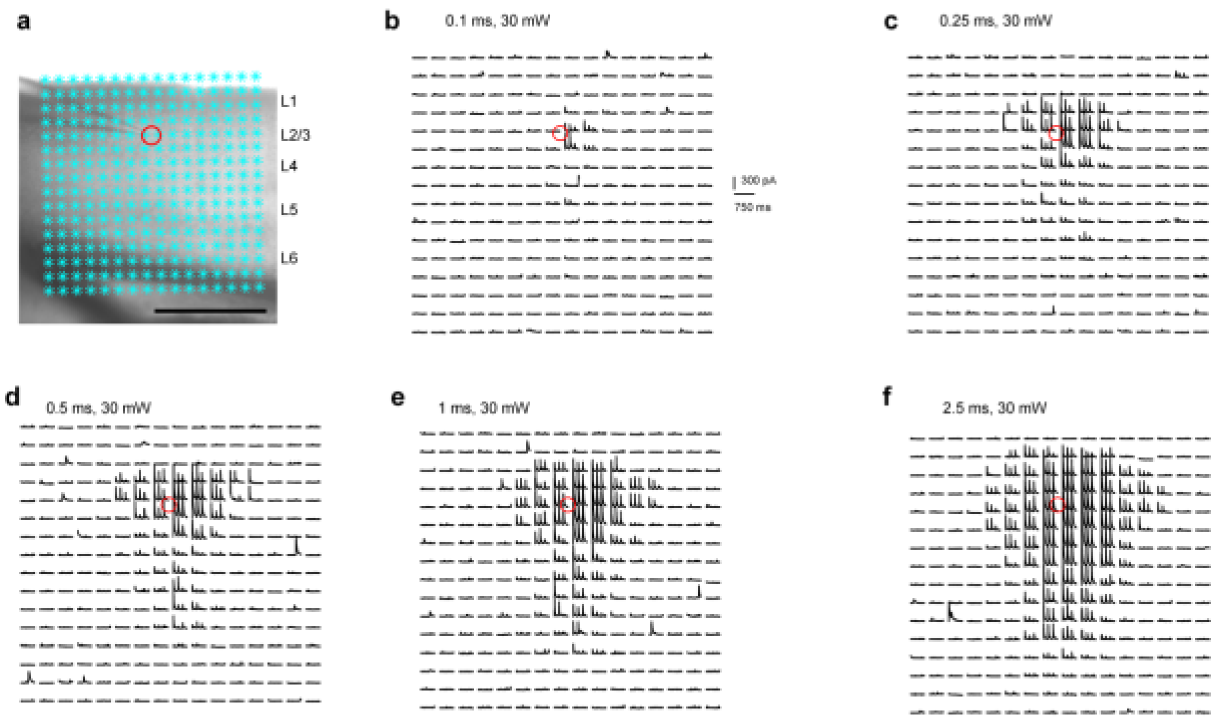

Supplementary Figure 2. Optogenetic stimulation mapping of PV specific inputs to a layer 2/3 excitatory pyramidal neuron at different laser pulse durations.

a. A representative mapping grid with ChR2 photoactivation sites (cyan *) is superimposed on the V1 slice image. The recorded pyramidal neuron's location is shown by the red circle. Layers 1-6 is indicated by L1-L6.

b-f. Plots of ChR2-evoked IPSC responses from pyramidal cell**.** Each panel shows responses evoked as a function of increasing laser pulse duration (0.1, 0.25, 0.5, 1 and 2.5 ms at 30 mW), respectively. Low laser power (0.1 ms duration) did not appear to robustly drive IPSCs from map locations outside the perisomatic region, while high laser power (e.g., 2.5 ms) lost laminar stimulation specificity as strong IPSCs could be evoked by L1 stimulation. L1 does not contain PV inhibitory neurons. Based on these measures, and the measures shown in Supplementary Figure 1, a photoactivation paradigm of 0.25 ms, 30 mW for used for subsequent mapping experiments.

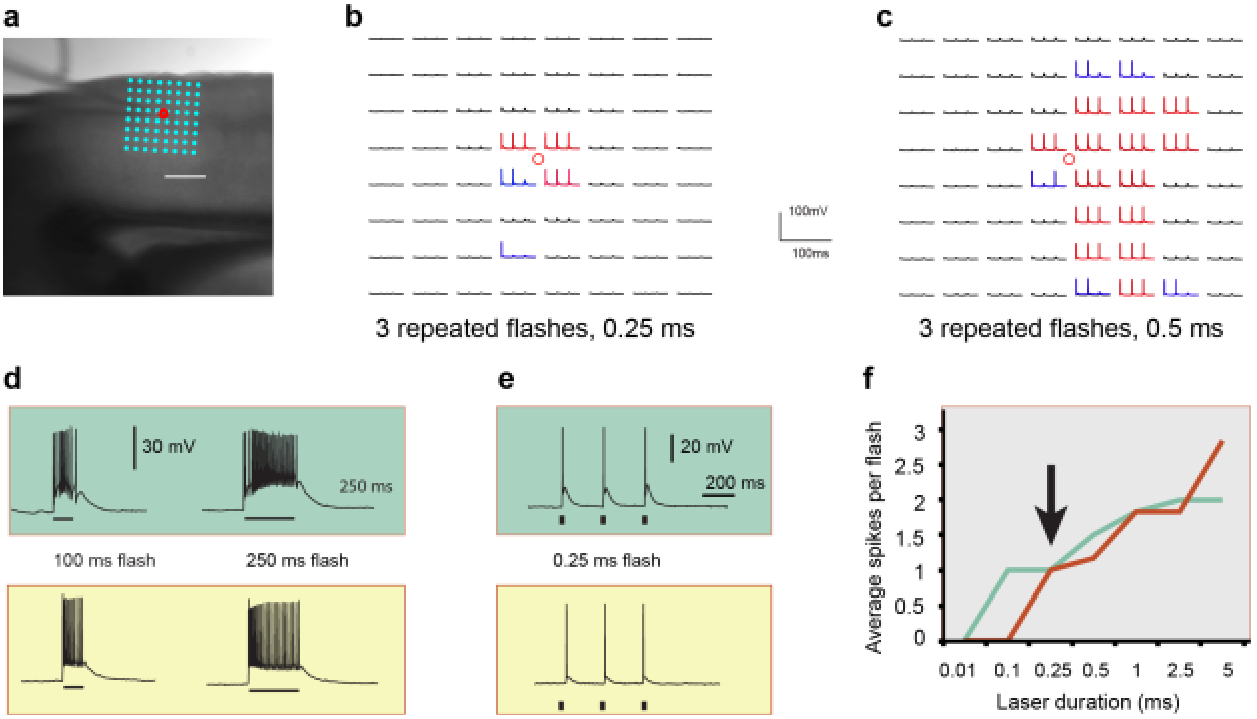

Supplementary Figure 3. Approach used to selectively stimulate PV cell somas using photostimulation of ChR2.

a. Infrared-differential interference contrast image of a coronal section through mouse visual cortex. A PV cell expressing ChR2 (position indicated by red circle) is patched by the micropipette seen coming in on the left side of the image. Blue dots show the position of blue laser light stimulation.

b. Responses from this cell to 3 very brief, 0.25ms laser light flashes at each of the 64 positions.

c. Responses of the same cell to 3 flashes of 0.5ms each.

d. Examples of spike trains elicited from normal PV cells (blue/green highlight) and PV cells hemizygous for Pten (yellow highlight) to a single 100ms laser flash and to a 250ms laser flash.

e. Examples showing the light stimulus and evoked responses used in the mapping experiments.

f. Measures of the average numbers of spikes evoked from normal (blue/green line) and mutant (orange line) PV cells at different laser durations. The arrow shows responses at 0.25ms, the duration used in these experiments.

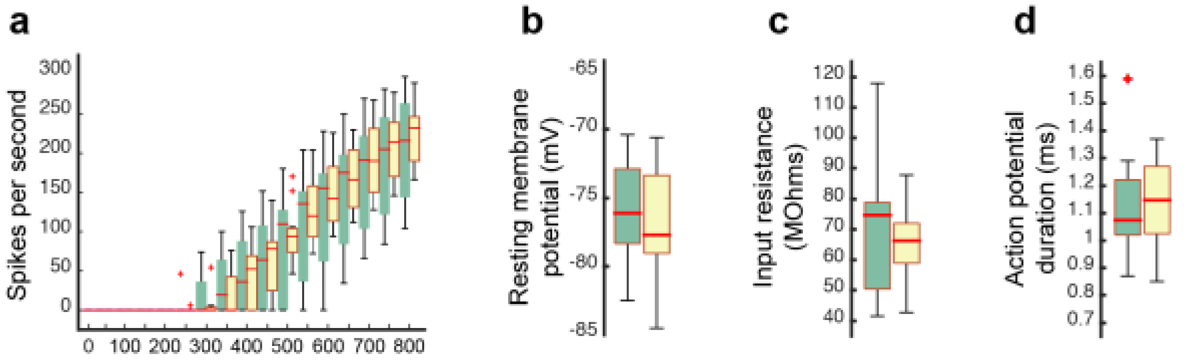

Supplementary Figure 4. Intrinsic membrane properties are not altered in PV cells hemizygous for Pten.

a. Boxplots of numbers of action potentials evoked in PV cells as a function of input current.
b. Boxplot of resting membrane potential.
c. Boxplots of input resistance.
d. Boxplots of action potential duration.
